# Neurofeedback Learning Is Skill Acquisition but Does Not Guarantee Treatment Benefit: Continuous-Time Analysis of Learning-Curves From a Clinical Trial for ADHD

**DOI:** 10.3389/fnhum.2021.668780

**Published:** 2021-06-29

**Authors:** Antti Veikko Petteri Veilahti, Levas Kovarskis, Benjamin Ultan Cowley

**Affiliations:** ^1^Department of Communication, Faculty of Humanities, University of Copenhagen Research Unit, Social Insurance Institution of Finland (Kela), Helsinki, Finland; ^2^NewPsy Institute, Helsinki, Finland; ^3^Faculty of Educational Sciences, University of Helsinki, Helsinki, Finland; ^4^Cognitive Science, Department of Digital Humanities, Faculty of Arts, University of Helsinki, Helsinki, Finland; ^5^Cognitive Brain Research Unit, Department of Psychology and Logopedics, Faculty of Medicine, University of Helsinki, Helsinki, Finland

**Keywords:** ADHD, adult ADHD, neurofeedback, EEG, clinical trial, learning, classification, continuous-time modelling

## Abstract

Neurofeedback for attention deficit/hyperactivity disorder (ADHD) has long been studied as an alternative to medication, promising non-invasive treatment with minimal side-effects and sustained outcome. However, debate continues over the efficacy of neurofeedback, partly because existing evidence for efficacy is mixed and often non-specific, with unclear relationships between prognostic variables, patient performance when learning to self-regulate, and treatment outcomes. We report an extensive analysis on the understudied area of *neurofeedback learning*. Our data comes from a randomised controlled clinical trial in adults with ADHD (registered trial ISRCTN13915109; *N* = 23; 13:10 female:male; age 25–57). Patients were treated with either theta-beta ratio or sensorimotor-rhythm regimes for 40 one-hour sessions. We classify 11 learners vs 12 non-learners by the significance of random slopes in a linear mixed growth-curve model. We then analyse the predictors, outcomes, and processes of learners vs non-learners, using these groups as mutual controls. Significant predictive relationships were found in anxiety disorder (GAD), dissociative experience (DES), and behavioural inhibition (BIS) scores obtained during screening. Low DES, but high GAD and BIS, predicted positive learning. Patterns of behavioural outcomes from Test Of Variables of Attention, and symptoms from adult ADHD Self-Report Scale, suggested that learning itself is not required for positive outcomes. Finally, the learning process was analysed using structural-equations modelling with continuous-time data, estimating the short-term and sustained impact of each session on learning. A key finding is that our results support the conceptualisation of neurofeedback learning as skill acquisition, and not merely operant conditioning as originally proposed in the literature.

## Introduction

Attention-deficit/hyperactivity disorder (ADHD) is a highly heritable condition with symptoms which begin in childhood and often continue into adulthood, and is currently estimated to affect 2.5–3.4% of the adult population (Kessler et al., 2006). Here, we report exploratory analyses of a randomised controlled clinical trial of neurofeedback treatment in adults with ADHD (registered trial ISRCTN13915109; Cowley et al., 2016), focusing on the understudied area of neurofeedback *learning*, and its implications for treatment.

In current diagnostic practice ADHD refers to substantial disability, of neurodevelopmental origin, in several dimensions of executive function: a “lack of persistence in activities that require cognitive involvement, and a tendency to move from one activity to another without completing any one, together with disorganised, ill-regulated, and excessive activity” (APA: DSM5, WHO ICD-10). Given the heterogeneous and comorbid profile of ADHD, psychostimulant treatments are not always specific or sustained (Monastra et al., 2002); and seem to have no effect for about a third of all ADHD patients (Hermens et al., 2006). In this context, neurofeedback (NFB) has been proposed as a complementary option for treatment of ADHD, with a rich literature and history of clinical application (Arns et al., 2014). In NFB, patients train self-regulation of certain features of their own neural activity, with the aim of reducing task-related attention deficits, and/or hyperactivity-impulsivity.

The clinical trial (Cowley et al., 2016) analysed in this study followed one of the most established approaches: patients must train to regulate the power (decibels) in specific bands of the electroencephalograph (EEG) frequency spectrum—e.g., theta-beta ratio (TBR) and sensorimotor rhythm (SMR) regimes target theta (4–8 Hz) and beta (13–30 Hz) bands. Other common approaches focus not on frequency bands but, e.g., on “slow” potentials of the EEG waveform (Strehl et al., 2017), or peripheral nervous system signals (Calderon and Thompson, 2004). It must be noted that in each case, the exact mechanism of the affected neural process is not fully understood. For example, in a recent study EEG spectral features were able to classify adult ADHD patients from controls, but TBR was *not* (Kiiski et al., 2020).

In summary, NFB refers to multiple regimes, which each have different partially understood effects, each potentially interacting in different ways with the subtypes of ADHD and the wide range of possible comorbidities; and delivered with limited treatment standardisation (Cortese et al., 2016). Therefore, the question “*is NFB efficacious for ADHD?*” is possibly ill-posed, as suggested by recent work promoting standardisation of NFB (Arns et al., 2020; Ros et al., 2020). The gold standard for efficacy assessment is a randomised controlled trial (RCT) with pre- vs post-treatment comparison of ADHD symptoms, but analysis at this level of detail has not provided a definitive conclusion. We must narrow down the broad question of efficacy to dig deeper into the component parts of the treatment. One neglected area of study, which is peculiar to NFB, is how well patients learn to perform the regulation task and how the quality of learning affects efficacy.

Thus far, most efficacy studies have failed to address the variability in NFB learning; that is, to distinguish between the “performers” and “non-performers” (Alkoby et al., 2017). This distinction is crucial because pooling data from subgroups of ADHD patients who benefit differently from NFB treatment, not only diminishes individual effect sizes (cf. Sonuga-Barke et al., 2013; Micoulaud-Franchi et al., 2014; Cortese et al., 2016), but also hides any qualitatively different effects manifested in different subgroups. Moreover, finding *reliable ways of identifying the performers would enhance prognosis and the allocation of clinical resources*.

We here report a novel, exploratory analysis of a clinical trial of NFB for adults with ADHD, focused on their learning and its predictors, correlates, and outcome effects. Our original research (e.g., sample size based on statistical power analysis, as detailed in section 2.5 of Cowley et al., 2016) was designed to test the *efficacy* of NFB training in the entire training group. We found no such general effect; however, (unlike data in most RCTs), our data includes detailed measurements from each training session, allowing us to distinguish between two groups of patients based on training progress. These data (and the consequent analysis approach which takes the *session* as the primary unit of observation, *N* ≃ 920) are sufficient to explore NFB learning: to characterise the learning process by linear mixed models and by continuous-time structural equations modelling of session-scores in a multilevel setting; and to test predictors and outcomes of learning. Despite the low sample size at the patient level, this study pioneers a demonstration of the complexity of NFB modelling, and makes a necessary and important contribution to guide the design of future NFB-related research.

### Aims

Our main aim is to analyse NFB learning, its predictors, and effects on ADHD behavioural outcomes. Part of the complexity of understanding NFB lies in the debate over what type of learning it represents and from what aspects of treatment the effects actually emerge. Traditionally, NFB training was viewed as *operant conditioning* aimed at “normalising” EEG and “repairing” some neurophysiological dysfunction (Gevensleben et al., 2009a). However, some recent research views NFB as skill learning (Strehl, 2014), aimed at learning a “tool for enhancing specific cognitive or attentional states in certain situations” (Gevensleben et al., 2009b: 781). The latter requires conscious processing, and there is evidence that skill-learning does not occur similarly in all patients in NFB training (Doehnert et al., 2008; Wan et al., 2014). Therefore, NFB cannot take place without a behavioural component [as noted in Schönenberg et al. (2017)], suggesting that it could be a form of *behavioural therapy* (Strehl, 2014). This has methodological implications for studying the efficacy of NFB training (Gevensleben et al., 2014), making it crucial to identify in advance those that can benefit (e.g., Truant, 1998).

Recent work has called for closer examination of NFB learning, both during training and in the long-term (Gevensleben et al., 2014; Zuberer et al., 2015). To our knowledge, the results presented here are the first to comprehensively analyse NFB learning in adults with ADHD.

Our prior paper (Cowley et al., 2016) focused on the protocol, and reported only the pre- to post-treatment ADHD symptom change compared to a wait-list control group. We found a treatment effect in placebo-confounded self-reports, but not in attention test scores. In this paper, we study NFB learning from the same data, exploiting three features of the trial’s design to help control for confounds due to the unclear mechanism of NFB.

**First**, the trial data was collected through all sessions, and self-reported symptom scores were collected four times for each patient.

**Second,** we used personalised NFB protocol assignment: after treatment-randomisation, patients were split between TBR and SMR training regimes, based on a ratio of theta to beta power greater than 1.

**Third**, we included “inverse training” trials in the set of sessions from halfway to the end of training. Inverse trials use feedback based on the inverse of the standard training target, e.g., regulating theta power up and beta power down.

### Research Questions

We address three overarching research questions (RQs).

First, **RQ1**: **how can we identify and characterize the learning observed in TBR and SMR regimes**? We model the magnitude of *gain in performance scores*, and classify patients into learner and non-learner groups based on model coefficient. To account for treatment scheduling variation, we also model how performance changes over *continuous* time during NFB training. Each model of the learning processes is examined for differences between TBR and SMR regimes.

Second, **RQ2**: **what clinically-relevant variables are *predictive* of likelihood-to-learn**? Prediction of NFB learning considers factors clinically-relevant but non-specific to ADHD. These factors include confounders of ADHD aetiology, such as trauma (Szymanski et al., 2011); and variance in biopsychological traits, such as behavioural inhibition, which have been shown to affect success in biofeedback training in a non-clinical sample (Kosunen et al., 2018). Finally, the *relative band power ratio (BPR)* is examined, as it was used as a basis of dividing patients into the two training groups.

Third, **RQ3**: **how do NFB learners differ from non-learners in terms of *treatment outcomes* for ADHD-related symptoms**? We examine whether outcome variables from self-report and behavioural ADHD-symptom tests differ between learner vs non-learner groups. Though it is not a research question of this paper, for illustrative purposes we include in Supplementary Material a contrast of learner and non-learner outcomes with the “untreated reference level” provided by wait-list controls (who recorded the same outcome data). No prior study has addressed whether positive NFB learning is a sufficient, let alone a necessary, condition for improvement of ADHD-related symptoms. We also examine whether any learning-predictive variables from RQ2 modified the effects of learning on outcome variables, contrasting learners and non-learners.

## Materials and Methods

Below we describe all methods used in this paper, but see Cowley et al. (2016) for details on the design and implementation of clinical trial ISRCTN13915109.

### Participants

Data here analysed come from 23 adults treated with NFB (13 females, 10 males; aged 25–57, m = 35.7, sd = 9.7). From an initial cohort of 54 screened volunteers, 26 patients were randomly selected for treatment: one dropped out during NFB training, and two (1 female, 1 male) were excluded from analysis due to insufficient data.

Inclusion criteria for the ADHD group were: (1) pre-existing diagnosis of ADHD/ADD, (2) no neurological diagnoses, (3) age between 18 and 60, (4) scores on Adult ADHD Self Report Scale (ASRS; Kessler et al., 2005) and Brown ADHD scale (BADDS; Brown, 1996) indicating the presence of ADHD, and (5) an IQ score of at least 80 using WAIS IV measured by a qualified psychologist (Wechsler, 2008). No strict cut-off values were used for ASRS and BADDS to indicate the presence of ADHD/ADD. Instead, exclusion was decided by the consulting psychiatrist, who conducted structured clinical interviews with participants using the Diagnostic Interview for ADHD in Adults (DIVA 2.0; Kooij, 2012). Comorbidities were evaluated during the clinical interview, and exclusion criteria included outlier scores in scores of Generalised Anxiety Disorder (GAD; Spitzer et al., 2006), Beck Depression Inventory (BDI; Beck et al., 1996), Alcohol Use Disorders Identification Test (AUDIT; Saunders et al., 1993), the Mood Disorder Questionnaire (MDQ; Hirschfeld et al., 2000), test of prodromal symptoms of psychosis (PROD; Heinimaa et al., 2003), and the Dissociative Experiences Scale (DES; Liebowitz, 1992). The psychiatrist followed DIVA guidelines to confirm the existing ADHD/ADD diagnosis, or not. All participants had normal or corrected-to-normal vision.

All patients were fully briefed about all study components, gave written informed consent for participation, and had access to a qualified psychiatrist. The study protocol followed guidelines of the Declaration of Helsinki for participants’ rights and study procedures. Approval was granted by the Ethical Committee of the Hospital District of Helsinki and Uusimaa, 28/03/2012, 621/1999, 24 \S. Participants were not remunerated.

### Procedure

Before NFB started, patients underwent eyes-open and eyes-closed baseline measurement of high-resolution 128-channel EEG in a shielded room. During this session we also obtained baseline behavioural and self-reported tests of ADHD symptoms (see section “Measures”). A similar session was applied after NFB treatment. Pre and post-treatment sessions had about 1 hour of task recordings each.

From the baseline EEG data, we computed individual spectrographs per condition and subtracted eyes-open from eyes-closed in order to determine the individual alpha peak frequency (IAPF; as per Lansbergen et al., 2011). We multiplied the canonical frequency band thresholds (e.g., 8 and 12 Hz for alpha) by the IAPF frequency to obtain individual frequency bands, and thus an individual theta/beta-ratio for assignment to TBR or SMR regimes, and individualised targets during NFB training. Among the 23 patients included in this study, 8 patients (3 female, 5 male) with theta/beta-ratio exceeding 1 were placed in the TBR training group. The other 15 patients (10 female, 5 male) received SMR training. SMR feedback was based on electrode C4 in the 10/20 system, whereas TBR feedback was based either on Fz electrode.

For the clinical trial (Cowley et al., 2016), we used automated constrained randomisation to assign patients to treatment or control group, so that groups had closely matched background variables such as age, IQ, diagnosis (ADHD/ADD), education, or gender. However, assignment to NFB regimes was not similarly controlled, so for the study reported here, background variables were tested between regime groups. The two groups did not significantly differ in any variables, although average age in the TBR group was 32.6 years, to 38.6 years in the SMR group, consistent with the finding that theta-beta ratio tends to decline with age (cf. Bresnahan et al., 1999).

**FIGURE 1:**
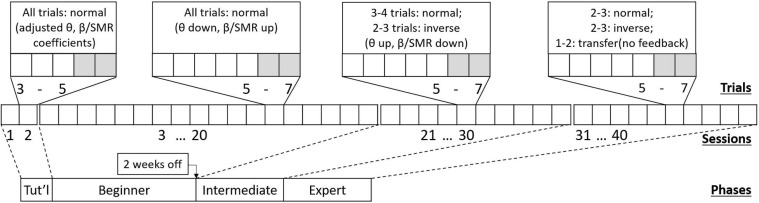
Schematic of the NFB treatment design, showing (from bottom to top) four phases containing 40 sessions with 5–7 trials each session. Each phase prescribes a different session-protocol, aiming to support patients learning to self-regulate.

NFB training was conducted in a professional clinical setting using an Enobio ambulatory EEG amplifier (Neuroelectrics SL, Barcelona). NFB treatment consisted of 40 training sessions (4 participants recorded only 39 usable sessions, and 1 only 38, due to technical issues), according to the design illustrated in Figure 1.

There were two to five sessions per week (max 1 session per day). Each lasted about an hour and typically consisted of 5–7 trials of NFB training. At the “*tutorial*” stage, that is, during the first two sessions, participants were given NFB trials that were made easier by adjusting baseline thresholds. During the first half of sessions (“*Beginner*” stage), only normal training trials were used. After this there was a mid-training break lasting up to two weeks. Following the break, during the second half of training (“*Intermediate*” stage), inverse training trials were introduced. Finally, in the last quarter of training (“*Expert*” stage), normal and inverse trials were accompanied by transfer trials (no immediate feedback given). As inverse and transfer trials were introduced, the number of normal trials was decreased to accommodate. Names given to stages are only descriptive, and not meant to imply a real level of skill—that would vary by individual.

### Measures

Measures fall into two types: study-level—calculated once per patient, e.g., learning curve slope; and session-level—calculated once per session, e.g., NFB performance averaged across trials in a session.

#### RQ1*—*Learning

To address RQ1 and estimate learning from performance data, during each session we recorded the average scores of normal, inverse, and transfer trials. The score of a trial was based on the percentage of time a patient achieved positive classification in the NFB task (e.g., in a normal TBR trial, this would be when theta band power is below and beta band power is above their respective baseline values). The scores were adjusted *post-hoc* to account for variance in baseline band powers, i.e., each trial score was multiplied by the baseline theta:beta ratio (or theta:SMR ratio) of that session. Inverse trials were baseline-adjusted by the reverse ratio. *We used normal trial scores from the first 30 sessions to classify NFB learning, thereby excluding transfer trials*.

#### RQ2*—*Prediction

To study the prediction of learning and address RQ2, two scales from the set of exclusion criteria (measured at screening) were used as “clinically-relevant” variables: Generalised Anxiety Disorder (GAD), and Dissociative Experiences Scale (DES). In addition, the Behavioural Inhibition Scale/Behavioural Activation Scale (BIS/BAS; Carver and White, 1994) was measured during the post-treatment lab session.

In addition, we calculated the BPR for each protocol. Specifically, for TBR patients the ratio was based on relative band power of theta vs full-spectrum beta at a frontal electrode. For SMR patients the band powers were recorded at central sites on a reduced bandwidth (12–15 Hz). Therefore, despite the different meaning of BPR under the two regimes, in both cases it still serves as a neurological marker targeted by NFB training, helping us to examine the pertinence of the operant conditioning framework.

#### RQ3*—*Outcome

To study the effect of learning on outcomes and address RQ3, we collected Test of Variability of Attention (TOVA, Greenberg et al., 2007) scores at pre- and post-NFB training; and Adult ADHD Self Report Scale (ASRS, Kessler et al., 2005) scores at pre-treatment and at sessions 10, 30, and 40. ASRS consists of 18 items tapping the frequency of recent DSM-IV criterion symptoms of adult ADHD, with sub-factors for Inattention (IA) and Hyperactivity-Impulsivity (HI). Also, TOVA provides measures of response time (RT), response time variability (RTV), omission errors (OM), commission errors (COM), the D-prime index of sensitivity to target vs. non-target stimuli, an index of symptom exaggeration, and an overall “attention performance index.” It has been argued that the set of behavioural TOVA scores provides an objective measure to indicate effectiveness of NFB training in terms of specific attentional properties such as impulse control and variability of response (Kaiser and Othmer, 2000). The internal consistency of TOVA scores has been verified (see e.g., Leark et al., 2004).

### Study Design

Statistical analyses were conducted by STATA version 14.2 except for continuous time structural equations modelling (Oud and Jansen, 2000), which was conducted by R version 3.4.1 and “ctsem”-package version 2.4.0 (Driver et al., 2015). In Results, we report all effects with exact *p*-values (where known), since our approach is exploratory and based on small sample sizes. Note that, as with measures, tests are defined either at study-level or session-level.

#### RQ1*—*Modelling NFB Learning

To address RQ1, we tackled three difficulties of modelling learning. **First**, the model of learning is unknown: literature currently cannot tell us which curve family or model, e.g., power law, exponential, or piecewise, would best apply to clinical NFB learning data. We addressed this by comparing the *heteroscedasticity* of different models. Following Wan et al. (2014)’s suggestion that the *logarithmic curve family* could be suitable to model non-clinical NFB learning, we applied linear mixed models (LMMs) to the logarithm of the training score, with patients as random-factor predictors, expressed as the following growth curve model:

$$\begin{array}{c} log\left(normalscore\right)=\beta_{x}x+\beta_{s}\times sessionnumber+u+error \end{array}$$

where level-two was formed by patients *x* and level-one by the session number *s*. Higher level variance component *u* was constant for each patient. We thereby obtained a model that was *homoscedastic*, i.e., error distribution of the model was independent of predicted score (see Supplementary Material for details). This log-transformed data also slightly improved the fitness of the model and helped to compress upper outliers.

The **second** difficulty is how to characterise the chosen components of learning: in our case, performance *gain*. Our estimate of gain is based on the absolute difference between intercept of the logarithmic LMM and value of the model at treatment end:

$$\begin{array}{c} gain=\left|Intercept-y_{t=30}\right| \end{array}$$

Then, we classified as “learners” those patients whose individual growth curve slope (*β*_*x*_) in the LMM was positive and statistically significant. This was tested by the Wald-test measure of the null hypothesis *β*_*x*_ ≤ 0; patients for whom this null could not be invalidated were classified as “non-learners.”

**Third**, there can be substantial variation in the learning effect across individuals. Variance in scores is handled using hierarchical models, as described; more difficult to manage is variance in the *temporal distribution of training sessions*. Despite starting with a uniform regular schedule, patients varied in how many sessions they had each week (due to cancellations or rescheduling for technical or personal reasons), and thus the amount of time between sessions varied. Since this is an unavoidable risk for clinical NFB trials (because even if sessions are strictly scheduled they might still fail due to uncontrollable factors), it is interesting to note that modelling such temporal variability *has never before been attempted*. Here, we analysed scores in context of the *continuous actual times* they occurred.

To do this, we studied the role of BPR and learning within each NFB regime, TBR and SMR, separately by a session-to-session process analysis of the evolution of group-means, based on structural equations modelling with continuous time data (CTSEM), using the R package “ctsem” (Driver et al., 2015). We particularly define it as a two-level model, where structural equations modelling is used to analyse the progression of different scores between sessions (level 1), clustered by patients (level 2). It should be noted that, in terms of statistical power, the number of level 1 units is more important and level 2 units are only used to account to between patient variance and differences in model coefficients. This is because the model is not used to identify between-patient differences or predictors, except for a comparison between TBR and SMR protocols. See the Supplementary Material for a technical description of the model.

The CTSEM models had three outcome variables: the BPR value, and the session averages for normal and inverse training scores. Moreover, because each training session has a specific impact on training scores, each session was incorporated as an exogenous predictor in two ways in order to estimate both its short-term impact (impulse) and its sustained effect (level change). The distinction between these two scenarios is important from the point of view of the learning process, because the consolidation of learning can be assumed to effectuate level change, whereas a short-term effect without long-term acquisition of skills would count as an impulse.

#### RQ2*—*Predict NFB Learning

RQ2 was tested using one-tailed t-tests of the difference between learner vs non-learners in scores from DES, GAD, and BIS/BAS. Because variables DES and GAD are highly correlated, their predictive value for identifying NFB learners was further assessed by forming three different linear probability models where either one of DES or GAD, or both, served as predictors of the classification of learners.

$$\begin{array}{c} P\left(learner\right)=\beta_{DES}DES+c_{D} \end{array}$$

$$\begin{array}{c} P\left(learner\right)=\beta_{GAD}GAD+c_{G} \end{array}$$

$$\begin{array}{c} P\left(learner\right)=\beta_{DES}^{\prime }DES+\beta_{GAD}^{\prime }GAD+c_{DG} \end{array}$$

At the within-subjects level, we tested the difference of learner vs non-learner BPR using one-tailed t-test. For this test each patient was ascribed with a variable *dBPR* that measures the change of the average BPR between sessions 1 to 5 and sessions 30 to 34. Further, we constructed a correlation table to test the mutual relevance of the variables: classification of learners, dBPR, DES, GAD and BIS.

Self-report variables collected at each session (e.g., effort, hours slept) were tested by a linear mixed model seeking to explain the logarithm of the normal session score:

$$\begin{array}{c} \log\left(\mathrm{normalscore}\right)=\beta_{x}x+\beta_{s}\times session+\beta_{interaction}\times session\times x+u+error \end{array}$$

where level-two was formed by patients and level-one by sessions (training days). Higher level variance component *u* was constant for each patient. The interaction coefficient *β_*interaction*_* was then tested for whether the specific predictor variable (e.g., mood or sleep) affected the slope of the learning curve. Moreover, other LMMs were built to address whether these variables affected scores at individual sessions:

$$\begin{array}{c} log\left(normalscore\right)=\beta_{x}x+u+error \end{array}$$

#### RQ3*—*Outcomes of NFB

To address RQ3, again we used one-tailed t-tests of outcome variables between learners and non-learners at single time points, or within-groups between two time points. For each TOVA variable, we calculated the differences of the pre- and post-training measures. For ASRS, we used the difference between the scores at the 30th session and before training.

Regarding the relationship of predictors with outcomes, and given the relevance of trauma in ADHD, we also classified patients by below median (<25) or above median (>=25) DES score before NFB training. Differences in the change of TOVA and ASRS scores were tested also for the high and low DES groups by using one-tailed t-tests. Moreover, in order to address the *specific contribution* of learning classification, DES, and BIS, we developed linear regression models with the specific TOVA measures as the outcome variables and three variables as the predictors (BIS, DES, and learner/non-learner status).

## Results

### RQ1

#### Identification of “Learners” and “Non-learners”

Based on the *gain* of the LMM of log-transformed session-wise NFB performance scores, we observed a robust learning effect across patients. We used standardised slopes of the LMM to split the sample between 11 learners and 12 non-learners. This classification is presented in Table 1, along with the main study-level variables such as clinical self-reports.

**TABLE 1:** Columns left-to-right: NFB regime; classification of learning; gain derived from the growth curve model of logarithmic scores over session numbers; z-transformed slopes of growth curve model; *p*-value of the model effect; patient’s BIS, DES, and BPR change values; classification of learning over sessions with inverse trials.

NFB regime	Age	Status	Gain (y_*t* = 30_-)	Standardised	Wald	BIS	DES	Change in	Inverse slope,
			intercept)	slope (z-score)	*p*-value			BPR	standardised
TBR	31	Learner	13.7	6.0	<0.001	19	9	0.16	1.0
TBR	32	Learner	8.8	4.5	<0.001	14	40	0.12	–0.8
TBR	37	Learner	14.0	3.6	<0.001	17	16	0.27	2.5
TBR	30	Learner	10.3	3.1	0.002	18	25	0.31	–1.3
TBR	33	Learner	6.4	2.3	0.02	14	12	0.12	0.1
TBR	30	Non-Learner	8.1	1.9	0.06	12	28	0.20	1.43
TBR	32	Non-Learner	0.1	1.6	0.12	16	64	0.04	3.3
TBR	37	Non-Learner	–1.2	–1.4	0.16	9	58	0.06	0.0
SMR	46	Learner	10.4	3.6	<0.001	16	4	0.08	–0.4
SMR	27	Learner	5.7	3.3	0.001	11	19	0.05	–0.3
SMR	29	Learner	12.1	2.7	0.007	11	59	0.10	1.7
SMR	55	Learner	5.5	2.5	0.01	15	44	0.13	0.4
SMR	31	Learner	6.4	2.3	0.02	16	51	–0.01	0.1
SMR	57	Learner	7.0	2.2	0.03	19	13	0.11	0.7
SMR	25	Non-Learner	10.0	1.3	0.19	8	7	0.12	0.3
SMR	38	Non-Learner	4.8	1.2	0.22	17	46	–0.17	1.2
SMR	46	Non-Learner	5.4	0.7	0.47	12	23	–0.13	–0.7
SMR	37	Non-Learner	2.7	0.6	0.55	14	22	0.22	1.0
SMR	28	Non-Learner	8.3	0.6	0.55	15	82	–0.04	–0.2
SMR	26	Non-Learner	2.2	0.3	0.73	13	91	–0.06	–2.0
SMR	37	Non-Learner	6.2	–0.7	0.49	7	118	0.03	–0.4
SMR	55	Non-Learner	–1.2	–1.4	0.17	16	22	–0.08	0.0
SMR	43	Non-Learner	9.0	–1.8	0.08	14	26	0.10	1.3

**FIGURE 2:**
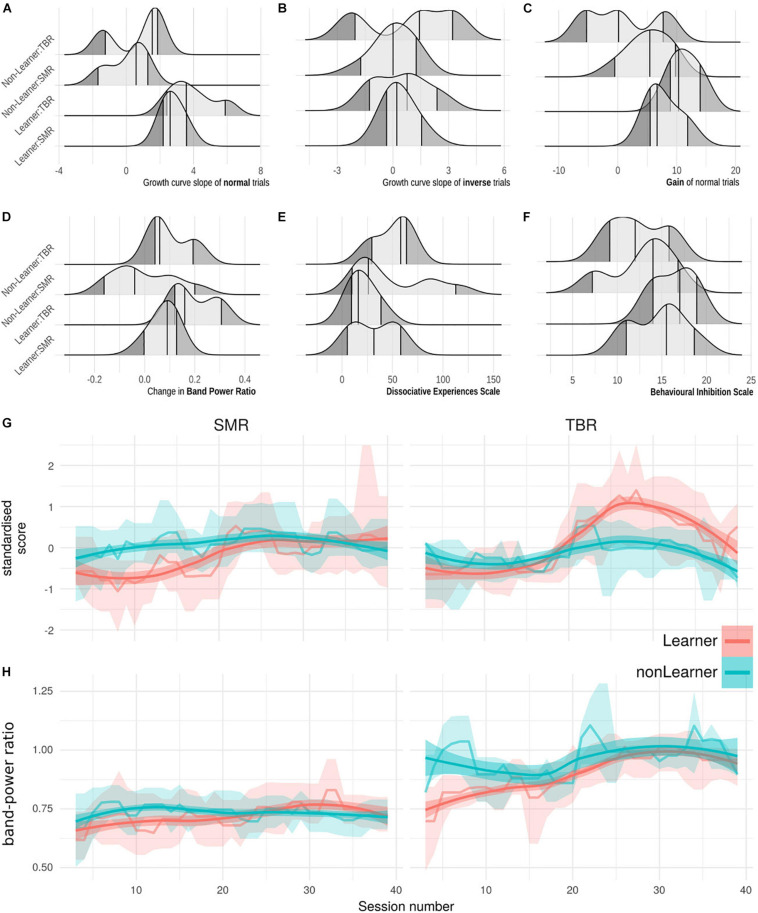
**(A–F)** Ridge plots of six learning-relevant variables (*x*-axes), grouped by categories of learning status and NFB regime (*y*-axes). **(A)** Slope of the growth model of normal-trial NFB performance, i.e., learning. **(B)** Slope of the growth model of inverse-trial NFB performance. **(C)** Gain in scores across NFB performance. **(D)** Change in baseline Band Power Ratio across treatment. **(E)** Behavioural Inhibition scale. **(F)** Dissociative Experiences scale. **(G)** Normal-trial performance scores at each session, grouped by learning status under TBR and SMR regimes. Lines are session-wise mean of all participant scores, bands are bootstrapped 95% CIs of the mean. Means and CI-edges are smoothed for visualisation with a running median of width 3. **(H)** Theta-Beta Ratio across sessions grouped by learning status and regime, as panel **(G)**.

Figure 2 (top) shows the distribution of key learning-related variables (slope of linear learning curves for normal and inverse scores, gain of normal scores, BPR, DES, BIS) for each combination of learning status and NFB regime. Figure 2 (bottom) shows the group means and bootstrapped 95% CIs of normal trial scores, smoothed, across all training sessions 3–40. At least in TBR regime, the last 10 trials show a downward trend which may relate to the reduced number of normal trials in per session averages, and/or a cross-over influence from the transfer trials.

#### Learning Over Continuous Time

**FIGURE 3:**
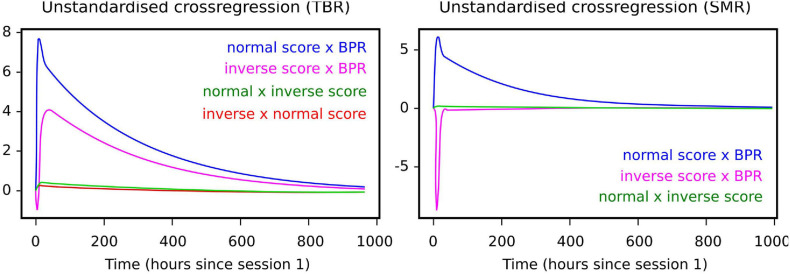
Cross-lagged effects (i.e., components of the drift matrix A) among learners under each NFB regime. Left panel: for TBR, cross-lagged effects indicate negative effect of inverse training scores on normal session scores. Right panel: for SMR, cross-lagged effects indicate positive effect of inverse training scores on normal training scores.

The CTSEM approach was used to specify how individual normal and inverse training trials affected NFB scores over continuous time. Figure 3 shows model outcomes among the learner group, demonstrating that NFB learning appeared to take place between four to eight days after a given training session. In other words, in the hypothetical situation that training was interrupted after a given session, it would be expected to take about one week before the full effect of past training sessions was visible.

Moreover, the cross-lagged effect of normal training scores on the BPR was visible among the learners under both NFB regimes (Figure 3), suggesting the possibility that the effects of NFB training are partly mediated by BPR changes.

When it comes to differences between regimes, inverse scores in the SMR regime also had a positive effect on the BPR, which means that inverse training is not necessarily harmful under the SMR regime. Under the TBR regime, by contrast, inverse training had a quick negative impact on normal training scores. The BPR itself did not have a notable negative effect on normal or inverse session scores. In particular, BPR is a lagging indicator whereas the normal and inverse training scores lead it. Comparing learning groups, for learners under both regimes the cumulative number of normal training trials appeared to have a positive learning effect on the normal score, whereas the non-learning groups did not demonstrate such effects.

### RQ2—Prediction of Learning

**FIGURE 4:**
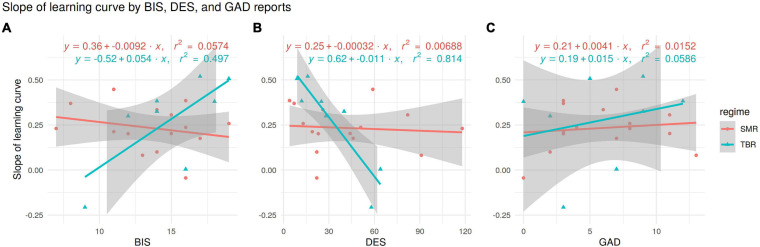
Slopes of patient-wise linear learning curves plotted against self-reports BIS **(A)**, DES **(B)**, and GAD **(C)**. Each panel shows separate linear regressions for NFB regime subgroups, annotated with equations and *r*^2^ values. The TBR regime subgroup shows strong positive relationship (*r* = 0.7) between BIS and learning curve **(A)**, and a negative relationship (*r* = −0.9) between DES and learning curve **(B)**.

The effect of DES, BIS, and GAD self-report scores on the learning slope is illustrated in Figure 4. DES has a negative correlation with the slopes of patient-wise learning curves (*r* = −0.35, *p* = 0.099), which appears to be driven entirely by the TBR group (Panel B), similarly as for BIS scores (though weaker with the opposite relation).

BAS scores of learners and non-learners did not differ significantly, but BIS scores did (BIS 15.4 [CI: 13.3–17.5] vs 12.8 [CI: 10.7–14.9], *t*(20) = −1.89, *p* = 0.04)—see Figure 4A.

#### Dissociative Experiences and Anxiety

Scores on the dissociative experiences scale (DES) were lower among the learner group (24.1 [CI 11.3–36.9]) than among the non-learners (46.3 [CI: 25.7–67.0], *t*(21) = 1.92, *p* = 0.04). The effect of DES scores on NFB learning was confirmed by a linear growth curve model, separately for both regimes (see Supplementary Material, Tables I.1, I.2).

Scores on the generalised anxiety disorder scale (GAD) were higher among the learners (7.1 [CI: 4.8–9.6]) than the non-learners (mean 4.8 [CI: 2.6–7.1]), though not statistically significant [*t*(21) = −1.46, *p* = 0.08]. GAD is also highly correlated with DES. Yet DES and GAD scores appear to have opposite effects on the learning status: it is those patients who score *high on anxiety but have low dissociative experiences score* that seem the most likely to be able to learn to self-regulate. When seeking to explain who were classified as learners, the model which combined both DES and GAD scores (Table 2) had higher F-score, and explained over twice the variance, compared to models with either DES or GAD as the only predictor.

**TABLE 2:** Linear probability models of the learning status as a function of DES, GAD or both, *N* = 23.

	Model I (DES)	Model II (GAD)	Model III (DES + GAD)
DES β-coefficients	−0.07*		−0.10**
GAD β-coefficients		0.035	0.065*
Intercept β-coef.	0.71***	0.25	0.45*
R^2^	0.157	0.065	0.351
F(1, 20)	3.72	1.40	5.14

*p < 0.05; **p < 0.01; ***p < 0.001. Learning status: non-learner = 0, learner = 1.

#### Baseline Band Power Ratio and Its Interactions

The learner/non-learner classification was not related to the initial BPR, but instead to the change between pre- and post-training BPR values. In particular, BPR was enhanced in the learner group (0.127 [CI: 0.064–0.191]), with no change among the non-learners (0.028 [CI: −0.050–0.107]), and the groups were significantly different [*t*(21) = −2.15, *p* = 0.02]. This suggests that changes in the BPR could serve as one mechanism mediating the effects of NFB learning. Thus we looked at its interactions with the aforementioned predictors of NFB learning—BIS, DES, and GAD scores. According to the following correlation table (Table 3), changes in the BPR (i.e., dBPR) were correlated particularly with DES scores (*r* = −0.35), whereas BIS values and GAD scores appeared to be associated with other learning mechanisms not directly associated with the BPR.

**TABLE 3:** Correlation table of different patient-level measures, *N* = 23.

	Learner	Change in BPR	BIS	DES
Change in BPR	0.42*			
BIS	0.39*	0.14		
DES	−0.40*	−0.35*	−0.42*	
GAD	0.26	–0.10	0.038	0.38*

*p < 0.05.

### RQ3—Outcomes of Learning

#### TOVA Behavioural Scores

Analysing the pre- to post-training change in behavioural scores from TOVA test, Cowley et al. (2016) found no statistical differences between the whole NFB treatment group and wait-list control group. In contrast, we found several effects comparing TOVA scores between learners and non-learners. The relationship between the three groups was typically that learners and non-learners changed in opposite directions, while wait list changed little, i.e., their scores lay between the other two groups.

Learner vs non-learner effects were: first, the gains in normal session scores were negatively correlated with the baseline measurement of omission errors, i.e., before NFB, learners made fewer omission errors than non-learners (learners 1.1 [CI: 0–2.55] vs non-learners 5 [CI: 0.16–9.83], *t*(20) = 1.568, *p* = 0.07). We compared the *change* of TOVA scores after training among the learners and non-learners. The omission errors appeared to become more common among the learner group [1.1 → 22.2, *t*(9) = 1.98, *p* = 0.04] while the effect was insignificant among non-learners [5 → 9, *t*(11) = 0.56, *p* = 0.30]. The wait-list group had approximately the same small degree of change as the non-learners (see Supplementary Material). Therefore, it is not obvious that NFB learning itself automatically leads to improvement in ADHD symptoms.

Learners also had higher pre-training D’ scores (i.e., the subject’s ability to discriminate the target stimulus from the non-target stimulus) than non-learners (learners 5.65 [CI: 4.81–6.49] vs. non-learners 4.74 [CI: 3.96–5.51], *t*(20) = 1.77, *p* = 0.05). Non-learners then scored higher *after* NFB training, though not significantly different to learners. In particular, the D’ score of learners declined from 5.65 to 4.86 [*t*(9) = –2.06, *p* = 0.03]; by contrast, the D’ scores of non-learners increased from 4.74 to 5.51 [*t*(11) = 1.5, *p* = 0.08].

The third difference between the learners and non-learners occurred in the change of response time variability, which increased among the learners [89.0 → 113.4, *t*(9) = 1.92, *p* = 0.04], but decreased among the non-learners [101.6 → 84.9, *t*(11) = 1.38, *p* = 0.10]. The group difference results are presented in Table 4. For both D’ and RTV, the wait list group experienced almost no change from intake to outtake, thus lying between learner and non-learner and not significantly different to either (see Supplementary Material).

**TABLE 4:** Average change of several TOVA values for learners and non-learners and *t*-values for testing differences learners and non-learners in relation to these changes.

	Non-learners	Learners	*t*	*df*	*p*
Change of standard score of D prime	0.77	–0.79	2.24	20	0.02
Change of response time variability	−16.7	24.4	–2.33	20	0.01
Change of omission errors	4	21.1	–1.48	20	0.08

#### ASRS Self-Report Scores

**FIGURE 5:**
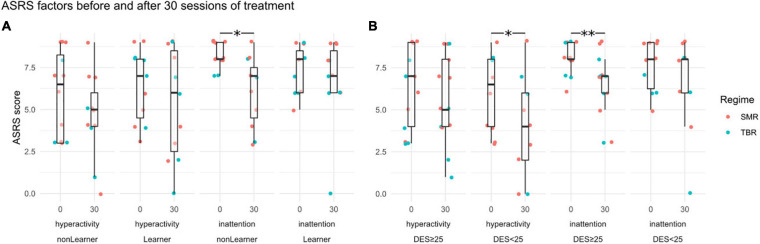
ASRS sub-factor scores (hyperactivity and inattention) before training at session 0 and at session 30. Panel **(A)** shows grouping by learners and non-learners, Panel **(B)** by DES < 25 and DES ≥ 25. Statistically significant differences between sessions 0 and 30 are indicated by asterisks (^∗^*p* < 0.05, ^∗∗^*p* < 0.01). Point-colouring by NFB regime reveals little difference.

Cowley et al. (2016) reported a significant decline in both ASRS factors—inattention and hyperactivity-impulsivity—for the treatment group compared with the control group. Within the treatment group, there was no difference between the learners and non-learners for hyperactivity [−1 vs −1.09, *t*(20) = 0.09, *p* = 0.93], whereas for inattention the difference between the two groups was just shy of statistical significance [−2.09 vs −0.55, *t*(20) = −1.65, *p* = 0.06]. This was driven by the fact that *inattention scores from the non-learners started higher and fell significantly*, as illustrated in Figure 5. The wait list group showed the opposite pattern, and had a significant increase of inattention scores compared to non-learners, but not compared to learners (see Supplementary Material).

By contrast, when it comes to confounding variables, the ASRS *hyperactivity* scores fell among those with DES value lower than 25 [5.82 → 3.80, *t*(9) = −1.95, *p* = 0.04], whereas there was no change in hyperactivity scores in the group with higher DES values [6.29 → 5.71, *t*(13) = −1.07, *p* = 0.15].

This is so even though the pre-training hyperactivity scores were uncorrelated with DES scores and the change in hyperactivity scores was independent of the improvement in NFB scores (which was a strong correlate of DES). The inattention scores, by contrast, fell more among those with DES values above 25 [7.79 → 5.93, *t*(13) = 3.36, *p* = 0.003], and not significantly among those with low DES scores [7.5 → 6.5, *t*(9) = 1.2048, *p* = 0.13].

#### Predictive Clinical Scores Modify Behavioural Effects

When controlling for the DES, BIS and GAD scores, which are strongly correlated with the classification of learners, it appears that those classified as non-learners still seemed to improve more than the learners in terms of D’—i.e., the ability to distinguish target and non-target stimuli—and response time variability, yet the effect was no longer statistically significant after control.

The effect of NFB learning on omission errors was virtually absent when BIS scores were controlled for. Higher scores in the dissociative experiences scale (DES), in contrast, seem to predict improvement in the attention performance index and D’ scores, despite the negative effect of high DES score on the learning status. In fact, D’ scores did not improve for those with DES values lower than 25 (−21.8 among low DES vs. 24.1 among high DES trainees, *t*(20) = 2.24, *p* = 0.02) and neither did the attention performance index [−2.9 low DES to 2.6 high DES, *t*(20) = −2.67, *p* = 0.01]. The *effect of training on TOVA scores thus appears to have been very different depending on whether the patient initially had low or high DES score* (see Table 5).

**TABLE 5:** Linear models of the change in several TOVA scores as outcomes, *N* = 23.

	Symptom	Attention	D′	Omission errors	Response time	Response time
	exaggeration index	performance index				variability
BIS	–0.9	–0.19	0.66	−2.14*	−2.08*	–0.69
DES	–2.13	1.75^+^	1.52	0.06	0.35	1.45
Learner	–0.23	−1.72^+^	−1.76^+^	–0.24	0.19	–1.28
Constant	10.48	–0.07	–0.63	1.58	1.58	0.33

^+^*p* < 0.10; *p < 0.05.

## Discussion

While the question of efficacy of NFB training has been extensively studied, few articles have dealt with the heterogeneity of NFB learning and whether NFB efficacy is dependent on NFB learning—despite that NFB is a treatment that is *supposed* to be learned! Such work is vital to identify how clinical background and NFB learning interact to produce beneficial treatment outcomes. Indeed, if some patients benefit more than others, the predominant focus of previous studies in the general effect of NFB on all ADHD patients, particularly when sample sizes are low, could be the reason why many meta-analyses have failed to identify a statistically significant effect (cf. Sonuga-Barke et al., 2013; Micoulaud-Franchi et al., 2014; Cortese et al., 2016).

This is the first study (that we are aware) to focus specifically on (a) how learning unfolds over the course of NFB training as modelled in uniform and in continuous time (RQ1), (b) whether there exist markers to identify *in advance* those patients who learn to self-regulate during NFB training (RQ2), and (c) how learners differ from non-learners in terms of the outcome effects of NFB training (RQ3). We have found substantive evidence regarding these questions and their interactions. Based on the learning process analysis using structural-equations modelling with continuous-time data, estimating the short-term and sustained impact of each session on learning, a key finding is that our results support the conceptualisation of neurofeedback learning as skill acquisition; not merely operant conditioning as originally proposed in the literature.

Significant predictive relationships were found in anxiety disorder (GAD), dissociative experience (DES), and behavioural inhibition (BIS) scores obtained during screening. Low DES, but high GAD and BIS, predicted positive learning. These results provide another source of evidence supporting the claim that NFB learning is skill acquisition, at least among those patients with higher BIS scores, because BIS scores were not strongly associated with changes in the BPR so that higher BIS scores might enable participants to self-regulate pertinent bands *in situ*. Finally, patterns of behavioural outcomes from Test Of Variables of Attention, and symptoms from adult ADHD Self-Report Scale, suggested that learning itself is not required for positive outcomes.

Though our work is exploratory and the sample size unfortunately small, if the results are supported by further studies, the implications are profound. In therapy research it is recognised that not all patients automatically benefit from a given type of therapy; treatment response is regulated by various factors due to both patient and therapist (Norcross and Wampold, 2011). High DES scores, for example, could indicate that patients suffer from trauma related symptoms rather than ADHD and thus might benefit less from NFB training. Even if the ADHD diagnosis is genuine, if NFB is not driven only by operant conditioning, then there could also be non-neural reasons why some patients fail to learn to self-regulate. These include psychological factors like “subjects” beliefs regarding their ability to gain control over technological devices,” or the lack of suitable mental strategies used in the learning process (Kober et al., 2013). For instance, a large portion of child ADHD patients exaggerate self-efficacy and ability (Owens et al., 2007), whereas low self-esteem seems to make learning slightly more effective (Newark and Stieglitz, 2010). Thus, psychoeducation of different strategies of processing NFB data could also be important, though challenging. The idea that NFB learning is skill acquisition also implies that motivational, attributional, and personality factors might play a stronger role (Gevensleben et al., 2014). Finally, increased knowledge of NFB learning not only benefits clinical applications but could also improve our understanding of neuroregulation and plasticity (Van Doren et al., 2017).

### RQ1—NFB Learning Modelled

Based on our growth-curve model of learning, we classified roughly half the patients as learners—this is exactly in line with previous studies of NFB learning (Doehnert et al., 2008; Wan et al., 2014). We also demonstrated the far more technically-challenging model of learning in continuous time, discussed in detail below.

One methodological consequence of our study, regarding modelling learning, is that linear growth curve models could lead to heteroscedasticity; instead, using the logarithm of the training score ensured homoscedasticity. Classification of learners and non-learners must be based on well-formed models; this is particularly important in designs like ours where the classification itself serves as a basis for further analyses.

The transformed data corresponds to a log-lin space (logarithm-transformed *DV* and linear *IV*), meaning the linear regression line of the model is an exponential curve in linear space—yet this result does not generalise. A thorough analysis of model fitting across all available NFB clinical trial data is called for, to establish if any general learning curve for NFB exists.

The CTSEM method revealed two key findings. First, under both TBR and SMR regimes, the BPR appeared to be affected by normal and inverse session scores, but inverse scores had a negative impact on the BPR *only under the TBR regime*. This suggests that the ability to up-regulate theta activity as indicated by high inverse session scores could have a negative impact on the TBR ratio in the following sessions. Second, under both regimes the BPR appears to be a lagging indicator whereas the normal and inverse training scores lead it, suggesting that our choice to use training scores rather than pre-session BPR scores as the basis of classifying learners is coherent with the data.

We also looked at the impact and sustained learning effects of individual training trials. Peculiarly, the non-learners appeared to receive an initially higher but non-lasting effect on normal scores under the TBR regime. A similar phenomenon was not observed under the SMR regime by using the CTSEM approach. Moreover, in both regimes the negative effect of inverse training on normal scores appeared to occur only among the learners, while inverse training appeared to be neutral among the non-learners. Inverse scores in our design were not independent of normal scores, which precluded their use as a predictor in other models; however, as a cross-lagged model, the CTSEM is capable of handling the effect of different training types on each other, and thus provides a valuable insight into the processes during training. Table 6 shows a summary.

**TABLE 6:** Effects visible among learners and non-learners under the two regimes based on CTSEM models.

	TBR		SMR	
	Non-learners	Learners	Non-learners	Learners
Normal training	Higher but non-lasting effect on normal scores	Long-lasting effect on normal scores	No visible short- or long-term effect on normal scores	Long-lasting effect on normal scores
Inverse training	No visible effect on normal scores	A negative effect on normal scores	No visible effect on normal scores	A negative effect on normal scores

### RQ2—NFB Learning Predicted

Learners and non-learners did not differ in most background variables: age, gender, verbal comprehension, perceptual processing or the initial band power ratio. They also did not differ in terms of motivation, mood or excitement measured before training.

The strongest finding is that DES scores are negatively related to learning (despite the fact that exceptional DES scores were used as an exclusion criteria for recruitment). Therefore, some patients with higher DES scores might have comorbid dissociative symptoms or they might even have been misdiagnosed with ADHD, their hyperarousal and inattention in fact stemming from trauma (cf. Szymanski et al., 2011). The results give tentative evidence that the studied NFB protocols are less efficient for dissociative patients. Interestingly, BIS scores also strongly and *positively related to learning*, indicating that variation of sensitivity in the aversion-regulation system might influence NFB learning.

These findings together show that TBR patients’ capacity to learn self-regulation was mediated by stronger aversion-regulation and less dissociative experience. The fact that we deliberately selected the TBR group by their baseline prognostic EEG features should imply that this group consisted of individuals for whom the TBR therapy would be more effective. However, the results of Kiiski et al. (2020) suggest that it is not so clear, since in their study TBR did not classify ADHD patients from controls, whereas EEG spectral features did. Kiiski and colleagues did not investigate their sample’s prognostic variables, thus the combined implication of our result and theirs might be that elevated TBR, a well-established finding among children with ADHD, is subject to maturation effects mediated by trait dissociation and/or aversion response.

### RQ3—NFB Learning Outcomes

We investigated the *effects* of NFB training, finding substantial but not uniform differences between learners and non-learners.

In terms of behavioural outcomes of NFB training, the results contrast interestingly with the initial assumption that the learner group would benefit more from NFB training than the non-learners. This result is somewhat counterintuitive and contrary to what we expected, demonstrating a positive effect of NFB training specific to the so-called “non-learners.” Unfortunately, the existing data does not allow us to speculate on the actual mechanism to explain this observation. However, it demonstrates that non-learners, too, are likely to make an effort during NFB training, with possibly different behavioural implications.

In effect, the behavioural outcomes of NFB training are not reducible to changes in NFB scores or the BPR, and the effect of training can be different in different patient groups. Part, but not all, of the effect on changes in the attention performance index and D′ scores are explained by higher DES scores. At the same time, BIS scores explained almost entirely the reduction of omission errors and response time among the learners. Therefore, it is interesting that NFB learning as such appeared to have no direct, general effect on behavioural scores but that they appeared to be mediated by correlates like DES, BIS etc.

In terms of self-report scores, there occurred a qualitative difference between the learners and non-learners; the latter group demonstrated a decline in inattention scores. Hyperactivity scores, though, declined for those patients with low DES scores (see Figure 5). The interaction of DES and learning status means it is not possible to tell whether improvement in NFB scores as such plays a part or whether, instead, it is the different bases of symptoms of the patients that explain the ASRS-related effects.

The most straightforward interpretation for why DES scores matter is that higher DES scores make participants less susceptible to changes in the BPR. In contrast, other predictors of training success like BIS and GAD appear to act through other mechanisms less related to changes in the BPR. Therefore, it is those *patients who have lower DES scores that are susceptible to alterations in the BPR and react well to NFB training*. In particular, the low-DES patients improved in ASRS hyperactivity scores.

### Contributions and Conceptual Implications

This study has made the following novel contributions:

1. This study identified a central role for the behavioural inhibition system and dissociative experiences in NFB training of ADHD patients, and supported the model of NFB learning as skill acquisition.
2. Several studies proposing a distinction between learners and non-learners have assumed that those classified as learners should benefit from NFB training (a view sympathetic toward the operant conditioning model), but our study has shown that also non-learners might benefit from NFB training.

Therefore, even if our results are far from being exhaustive in regard to the specificity of NFB training on ADHD patients, the observed differences between learners and non-learners provide a clear indication that previous NFB research, which has mainly focused on studying the difference in pre- and post-training group averages, is limited in two regards. First, learning takes place over the sessions and it is not tied to the improvement of training scores or the BPR measurements as would be assumed by the operant conditioning model. The fact that the learners were slower to adapt to NFB training but that the effects were sustained in comparison to the non-learners suggests that there are possibly multiple neural networks involved in NFB training and multiple overlapping ways to conceptualise NFB learning. NFB learning is thus a complex question, mirroring also the complexity of the concepts of hyperactivity and inattention. These results also reinforce the view that NFB training should be viewed as a form of behavioural therapy (Strehl, 2014).

### Limitations and Future Work

The small sample size—though comparable with most NFB studies, due to their resource intensive nature—is the main limitation of this study, meaning many results are necessarily tentative. Also, the study populations under the two NFB regimes differed depending on their initial TBR-value, although this was a clinically necessary feature and for the sake of (most of) our statistical analyses, was considered as a single, personalised protocol. Moreover, in our study, inverse and the so-called transfer trials were introduced in the latter half of the study, and while we have not included the data except for inverse trials when developing the CTSEM models, the trials themselves could have affected also the outcomes measures, e.g., the strong decline in performance observed at least in TBR learners (cf. Kleinnijenhuis, 2007). However, despite these limitations, it is safe to conclude that the benefits of NFB training vary across different patient groups.

Methodologically, we chose to use a dichotomy of learning, instead of using the standardised model slopes directly in our analyses, though the latter have more information. This was due to the small sample size: future work on large samples would be free of these constraints. This methodological choice also allowed us to render the discussion of results in terms of learners vs non-learners, to provide clearer statements.

Future research must also assess whether observed differences are due to different techniques by which patients approach NFB training, or whether variables like DES and BIS reflect differences in patients’ neural capabilities and deficits. In particular, the link between BIS and NFB learning, observed now in Kosunen et al. (2018) and out study, should be further explored. Also, future research should compare the neurological bases of inverse and normal training across the NFB regimes.

As mentioned in the Introduction, the appropriate model of learning to use for NFB is not known—NFB is not a visuomotor task that can be assumed to follow power law curves. Large scale modelling of learning is thus required to establish an empirical picture of the shape of NFB learning. Although not a clinical study, Kovacevic et al. (2015) performed an interesting analysis of a suitably large sample (*N* > 500) of NFB learners, albeit in a single recording (not longitudinal). Their study complements our results in the sense that both hint at what can be achieved by learning analyses. Future work should examine the records of prior clinical trials of NFB for ADHD, thus to combine large N with longitudinal data.

This point also bears on the popular notion that sham NFB would provide a gold standard of control. While more studies *should* be conducted using sham NFB to test the question of efficacy (though see Pigott et al., 2021), it is not so clear when it comes to learning. In the sham control condition itself, patients would not exhibit real NFB performance, and so an artificial performance learning curve would have to be “programmed in” to the condition. All comparisons to a real treatment would then depend on this programmed learning curve.

## Conclusion

Existing evidence for the efficacy of NFB training on reducing ADHD core symptoms is mixed and non-specific, with differing effect strengths for different NFB regimes and even the different sub-types of ADHD. The results of this study have shown that important predictors of NFB learning, at least in the context of TBR training, are low dissociative experiences score and high behavioural inhibition score. At the same time, the likelihood of NFB learning is enhanced by elevated generalised anxiety disorder score. In addition, the gains in NFB scores do not appear to be a necessary condition for positive behavioural or self-reported effects, which could instead derive from the mere attempt to self-regulate. Our sample size was limited, so more research must be done to understand the apparently different neurological mechanisms of NFB training among the learners and non-learners, and the connection of these mechanisms to its qualitatively different effects on inattention and hyperactivity.

## Data Availability Statement

The raw data supporting the conclusions of this article are available from the figshare repository: https://figshare.com/projects/Adult_ADHD_Neurofeedback_trial/116040.

## Ethics Statement

The studies involving human participants were reviewed and approved by Ethical Committee of the Hospital District of Helsinki and Uusimaa, 28/03/2012, 621/1999, 24\S. The patients/participants provided their written informed consent to participate in this study.

## Author Contributions

AV conceived and conducted the analysis. LK contributed to the trial design and implementation. BC designed and implemented the trial, curated the data, and created the figures. All authors contributed to writing the manuscript.

## Conflict of Interest

The authors declare that the research was conducted in the absence of any commercial or financial relationships that could be construed as a potential conflict of interest.
